# Blebs—Formation, Regulation, Positioning, and Role in Amoeboid Cell Migration

**DOI:** 10.3389/fcell.2022.926394

**Published:** 2022-07-15

**Authors:** Jan Schick, Erez Raz

**Affiliations:** Institute of Cell Biology, ZMBE, Münster, Germany

**Keywords:** bleb, amoeboid migration, cell polarity, cell migration, actin, retrograde flow, myosin

## Abstract

In the context of development, tissue homeostasis, immune surveillance, and pathological conditions such as cancer metastasis and inflammation, migrating amoeboid cells commonly form protrusions called blebs. For these spherical protrusions to inflate, the force for pushing the membrane forward depends on actomyosin contraction rather than active actin assembly. Accordingly, blebs exhibit distinct dynamics and regulation. In this review, we first examine the mechanisms that control the inflation of blebs and bias their formation in the direction of the cell’s leading edge and present current views concerning the role blebs play in promoting cell locomotion. While certain motile amoeboid cells exclusively form blebs, others form blebs as well as other protrusion types. We describe factors in the environment and cell-intrinsic activities that determine the proportion of the different forms of protrusions cells produce.

## Introduction

Cell migration is a key process in development, immune response, and tissue homeostasis. This process is tightly regulated, and defective migration can result in clinical consequences such as cancer metastasis and chronic inflammation. Cells migrate as single cells or as a group, and these types of cellular movement can be further subdivided based on the precise mechanisms that facilitate the movement. Here, we focus on cells such as immune cells, metastatic cancer cells, and germ cells that migrate as single cells, physically independent of one another (Friedl and Wolf, 2003; Raz, 2004; Hind et al., 2016; Grimaldi and Raz, 2019). Based on morphology and protrusion types, the migration of single cells can be subcategorized into mesenchymal and amoeboid. Mesenchymal migration is characterized by thin, sheet-like protrusions called lamellipodia at the front of the cell (Abercrombie et al., 1970; Rottner and Schaks, 2019). In lamellipodia, arrays of branched actin filaments push the plasma membrane, thereby contributing to the translocation of the cell in a specific direction. By contrast, amoeboid (*ἀμοιβή* (Greek) = transformation, change) migration is characterized by rapid changes in cell morphology (Lämmermann and Sixt, 2009; Fritz-Laylin et al., 2018). Amoeboid cells are globular, exhibit a high degree of deformability, and form different protrusion types during their migration.

A distinct protrusion type generated by amoeboid-motile cells are spherical membrane bulges termed blebs. These protrusions are devoid of F-actin within the protrusion itself and depend on non-muscle myosin II-mediated (hereafter referred to as myosin) contractility that generates hydrostatic pressure (Charras and Paluch, 2008; Paluch and Raz, 2013). However, motile amoeboid cells can also form protrusions that are powered by active actin assembly, such as pseudopodia observed in leukocytes and *Dictyostelium discoideum* (Lämmermann and Sixt, 2009).

**FIGURE 1:**
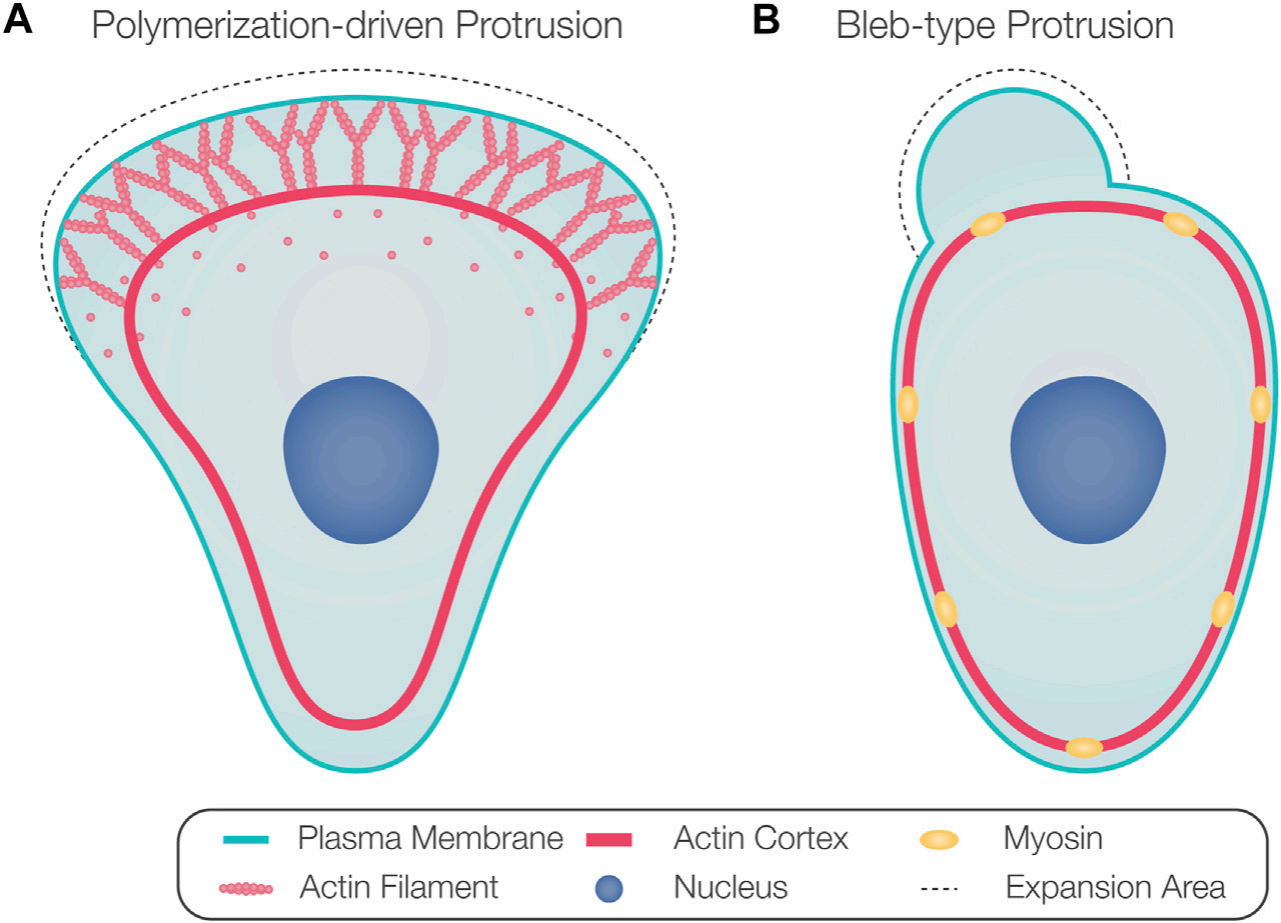
Protrusion formation mechanisms. **(A)** Arrays of polymerizing actin filaments pushing against the plasma membrane generate force to drive membrane protrusion forward. **(B)** Actomyosin contraction generates hydrostatic pressure that powers the inflation of a spherical membrane bleb.

Thus, classifying cell motility based on cell and protrusion morphology does not necessarily represent the underlying mechanism of protrusion formation. Therefore, we refer to protrusions powered by active actin assembly as *polymerization-driven protrusions* (Figure 1A) and protrusions whose extension depends on actomyosin contractility-generated hydrostatic pressure as *blebs* (Figure 1B). While polymerization-driven protrusions have been well described in cell motility, blebs are less understood. Blebs were originally considered hallmarks of apoptosis and necrosis (Laster and Mackenzie, 1996) and were later recognized as a common protrusion type formed by migrating cells, especially in three-dimensional (3D) environments (Charras and Paluch, 2008; Fackler and Grosse, 2008; Paluch and Raz, 2013).

Here, we review the current literature on bleb-driven motility. Specifically, we describe the generation of blebs, the mechanisms that direct these protrusions to the leading edge of migrating cells, and the factors that promote their formation, as distinct from polymerization-driven protrusions. Finally, we discuss the possible roles of blebs in migration.

## Formation and Regulation of Blebs

Blebbing activity can be divided into three stages: initiation, growth, and retraction (Charras and Paluch, 2008). Bleb initiation occurs as the plasma membrane starts separating from the underlying actin cortex, whereas during the growth phase, the flow of actin-free cytoplasm leads to further detachment of a larger membrane area from the cortex (Cunningham, 1995). The growth of blebs occurs rapidly on the timescale of tens of seconds (Charras et al., 2008). For example, in *Dictyostelium,* the leading edge of the cell advances at speeds of up to about 2.5 μm/s in the case of blebs, while polymerization-driven protrusions advance at speeds lower than 1 μm/s (Zatulovskiy et al., 2014). Bleb growth was found to depend on the unfolding of membrane invaginations, which increase the apparent membrane area and allow for inflation of the bleb (Goudarzi et al., 2017). As actomyosin contractility is required for bleb formation, inhibiting myosin function interferes with bleb generation (Yoshida and Inouye, 2001; Blaser et al., 2006; Goudarzi et al., 2012). Key activators of myosin contractility are RhoA, the downstream effector Rho-kinase (ROCK), and myosin light-chain kinase (MLCK) (Ridley, 2001). Accordingly, in the context of bleb-based motility, RhoA/ROCK signaling has been shown to drive the formation of the protrusions, while Rho GTPases belonging to the Rac subfamily facilitate actin assembly at the base of the bleb and also drive the extension of polymerization-driven protrusions (Sanz-Moreno et al., 2008; Ridley, 2015).

By measurements of the number and size of blebs in experimentally manipulated cells, bleb formation was found to depend on hydrostatic pressure. For example, it was observed that the volume of an individual bleb decreased when multiple consecutive blebs formed or when the pressure difference between the inside and the outside of a cell was reduced by electroporation (Tinevez et al., 2009; Maugis et al., 2010).

Similarly, the role of hydrostatic pressure and cytosolic flows in bleb inflation is in agreement with the finding that blebbing cells maintain their volume, as bleb inflation at the cell front results in a concomitant retraction of the cell back (Charras et al., 2005; Goudarzi et al., 2017, Goudarzi et al., 2019). Towards the end of bleb expansion, the actomyosin cortex reforms underneath the plasma membrane, and its contraction can drive the retraction of the bleb or the formation of a consecutive bleb (reviewed in (Charras and Paluch, 2008; Ikenouchi and Aoki, 2021)). In some cases, cells form a large leading edge bleb that does not undergo retraction during migration (Liu et al., 2015; Logue et al., 2015; Ruprecht et al., 2015). This phenomenon has been termed “leader” or “stable” bleb-based migration (hereafter referred to as “stable bleb migration”).

**FIGURE 2:**
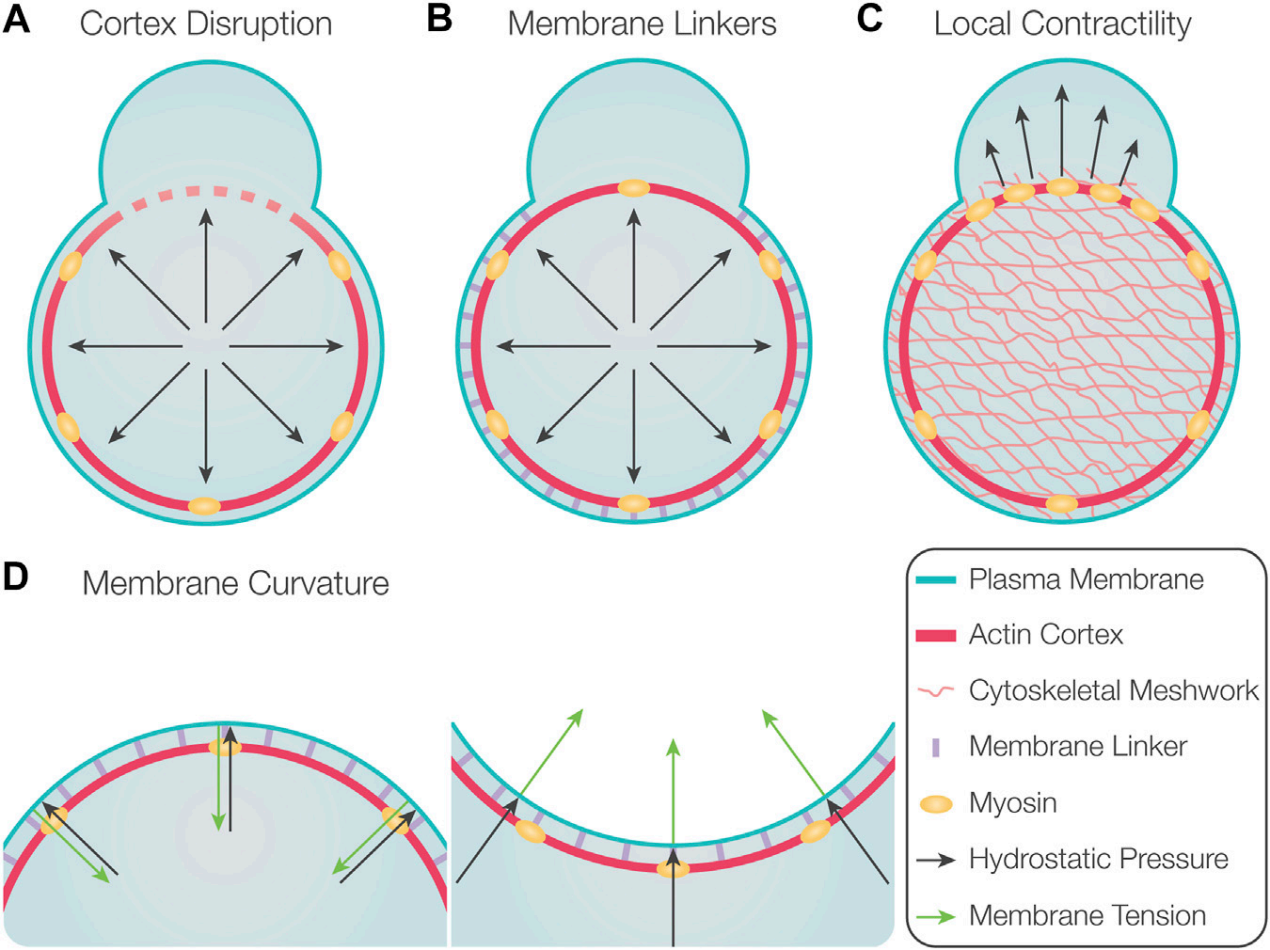
Factors controlling the position of bleb formation. **(A)** Global hydrostatic pressure induces bleb formation at locations where the actin cortex is disrupted. **(B)** Preferential initiation of blebs at locations with reduced levels of membrane linker molecules. **(C)** Locally increased contractility could induce bleb formation at these loci if poroelastic properties of the cytosol prevent rapid pressure equilibration. **(D)** Depending on membrane curvature, the force resulting from membrane tension is either directed inward in the case of positive membrane curvature (left) or outward for negative membrane curvature (right). Therefore, membrane delamination occurs more readily at locations of negative membrane curvature.

An especially interesting and important issue is the mechanisms that dictate the position at which the blebs form. In general, blebs are more likely to initiate at points where the degree of interaction between the cortex and the membrane is lower. Experimentally, this parameter can be modulated in several ways. First, blebs can be induced by locally disrupting the actin cortex (Figure 2A), such as in experiments by using laser ablation (Sedzinski et al., 2011; Goudarzi et al., 2012) or treatment with drugs that lead to actin depolymerization (Paluch et al., 2005; Sedzinski et al., 2011).

Second, the actomyosin cortex is typically connected to the plasma membrane by specific linker molecules, such as members of the ERM (ezrin, radixin, and moesin) family (Fehon et al., 2010). These molecules can inhibit the initiation and inflation of blebs because they suppress the initial detachment and further separation of the membrane from the underlying cortex (Figure 2B). Indeed, overexpression of ezrin results in reduced bleb formation, and, conversely, impairing ezrin activity leads to an increase in blebbing (Charras et al., 2006; Olguin-Olguin et al., 2021). Likewise, blebbing can be increased by interfering with the activity of ERM molecules and by altering the composition of lipids they bind on the plasma membrane (Dumstrei et al., 2004; Diz-Muñoz et al., 2010). Additional membrane linkers that control the formation of blebs are annexin and talin (Wang et al., 2008; Goudarzi et al., 2012; Tsujioka et al., 2012).

Third, the position of bleb initiation was suggested to be dictated by local contractility and an elevation in local pressure, which leads to initiation and inflation of the bleb (Figure 2C) (Charras et al., 2009). This proposed mechanism relies on the assumption that the hydrostatic pressure does not equilibrate instantaneously throughout the cytosol, such that a local elevation of pressure can be maintained for sufficient time. For this to be possible, the cytoplasm should possess poroelastic properties that reduce the speed with which pressure differences equilibrate within the cell (Charras et al., 2009). This model is supported by observations in melanoma M2 cells, where preventing the formation of blebs in one region of the cell did not affect the formation of blebs at other locations (Charras et al., 2005). Yet, for these poroelastic effects to be relevant for preventing fast pressure equilibration, the pore size needs to be sufficiently small, a factor that could markedly differ among different cell types (Mitchison et al., 2008). Indeed, in L929 fibroblasts and *Entamoeba histolytica*, the inflation of a bleb reduces the formation of subsequent blebs globally, indicating rapid equilibration of intracellular pressure (Tinevez et al., 2009; Maugis et al., 2010). A recently suggested mechanism for promoting blebbing is an increase in fluidization of cytosol within the forming protrusion, as compared with the more viscous properties of the cell body (Aoki et al., 2021).

Last, other studies have indicated that the position at which blebs are initiated is influenced by the local curvature of the plasma membrane, as regions of higher negative curvature were found to harbor more blebs (Figure 2D) (Maugis et al., 2010; Tyson et al., 2014). This is likely because the force resulting from membrane tension is directed inward in regions with positive curvature, whereas in regions of negative curvature, the force is directed outward (Tyson et al., 2014; Collier et al., 2017). Consistently, blebs are readily formed at the flanks of protrusions, regions that exhibit high negative curvature (Tyson et al., 2014). Based on this model, structures such as filopodia or actin microspikes could also favor bleb formation due to the high negative curvature they induce. Accordingly, studies in *Dictyostelium* and zebrafish primordial germ cells (hereafter, PGCs) showed that sites containing microspikes and filopodia exhibit increased blebbing activity (Zatulovskiy et al., 2014; Meyen et al., 2015).

## Biasing Bleb Formation to the Leading Edge

To effectively migrate, motile cells need to bias the formation of protrusions to the leading edge and retract at the opposite aspect of the cell. Below, we present the mechanisms that orient the formation of blebs to the leading edge and suppress their formation at the back.

Because the sites at which blebs form are often devoid of membrane linker molecules, motile cells may bias bleb formation to the leading edge by accumulating linker molecules at the rear (Collier et al., 2017; Olguin-Olguin et al., 2021). Indeed, elevated levels of the protein ezrin were observed at the rear of different bleb-driven motile cell types such as Walker 256 carcinosarcoma cells (hereafter, Walker cells) (Rossy et al., 2007), melanoma cells (Lorentzen et al., 2011), zebrafish PGCs (Olguin-Olguin et al., 2021), and zebrafish mesodermal progenitors (Ruprecht et al., 2015). Similarly, the linker protein talin has been found to accumulate at the rear of bleb-motile *Dictyostelium* cells (Collier et al., 2017). Other proteins reported to be localized to the back of bleb-driven zebrafish PGCs are the plasma membrane-endoplasmic reticulum connector extended synaptotagmin-like 2a and Septin9a, which could inhibit the separation of the membrane from the cell body and reinforce the actin cortex (Olguin-Olguin et al., 2021).

To enable directional locomotion, the molecules listed above need to be translocated toward the rear of the cell. A possible mechanism for translocating the bleb-inhibiting linker molecules to the cell back is actin retrograde flow. Actin retrograde flow occurs when polymerizing actin pushes against the front of the cell and, simultaneously, myosin contracts, which causes the actin filaments to flow towards the rear (Mitchison and Kirschner, 1988; Henson et al., 1999; Pollard et al., 2000; Babich et al., 2012). In zebrafish PGCs, actin retrograde flow was found to be prevented by inhibiting myosin contractility or actin polymerization, which, in turn, abrogated the polar distribution of membrane linker molecules (Grimaldi et al., 2020; Olguin-Olguin et al., 2021). Conversely, a strong increase in contractility results in robust polarization and the formation of a stable-bleb front devoid of linker molecules (Ruprecht et al., 2015; Olguin-Olguin et al., 2021).

Other studies have further suggested that leading-edge blebs may be oriented by polarized actomyosin contractility since especially high levels of myosin have been observed at the leading edge of migrating cells (Gutjahr et al., 2005; Blaser et al., 2006; Rossy et al., 2007; Grimaldi et al., 2020; Gabbireddy et al., 2021). Consistently, MLCK, activated MLC, and RhoA have been detected at the leading edge of migrating zebrafish PGCs (Blaser et al., 2006; Kardash et al., 2010). This distribution of myosin and its activity could result in breaks in the actin cortex preferentially at this region of the cell, thereby favoring the formation of blebs at the front (Paluch et al., 2005; Paluch and Raz, 2013).

Furthermore, polarized contractile activity could also result in an unequal distribution of intracellular pressure, favoring the inflation of blebs in the migrating cell at the region where contractility is elevated (Charras et al., 2005). However, certain migratory cell types that form blebs exhibit rearward localization of the contractile machinery, suggesting that local pressure elevation is not always essential for driving leading-edge bleb formation. For instance, *Amoeba proteus*, melanoma cells, and stable bleb-forming cells show increased myosin levels and activity at the back (Stockem et al., 1982; Pinner and Sahai, 2008; Liu et al., 2015; Ruprecht et al., 2015).

Increased contractility was also reported to cause the rearward movement of E-cadherin, indicating that this cell-cell adhesion molecule is also advected by actin retrograde flow (Kardash et al., 2010). Notably, one study showed that the engagement of E-cadherin with actin can contribute to polarizing bleb formation (Grimaldi et al., 2020). Here, E-cadherin molecules that engage with E-cadherin molecules of neighboring cells and actin within the cell itself may generate friction that inhibits actin retrograde flow. The organization and interactions among these structural proteins contribute to focusing actomyosin contractility and, therefore, the formation of blebs to the cell front (Grimaldi et al., 2020). Together, these findings show that different strategies can act in directing bleb formation to the leading edge.

Another interesting aspect of their polarization is how bleb-motile cells specify the front-back axis in response to guidance cues. In the case of zebrafish PGCs, migration is guided by the chemokine Cxcl12a, such that the cells polarize, form blebs toward the high end of the gradient, and migrate in the same direction (Doitsidou et al., 2002; Boldajipour et al., 2008; Olguin-Olguin et al., 2021). Similarly, *Dictyostelium* cells polarize, form blebs, and migrate in the direction of the source of a chemoattractant, cyclic AMP in this case (Heid et al., 2005; Langridge and Kay, 2006; Yoshida and Soldati, 2006). Such extracellular guidance cues activate their cognate receptors, G-protein coupled receptors (GPCRs), Cxcr4b, and cyclic AMP receptors in the examples presented above (Insall et al., 1994; Doitsidou et al., 2002). The ligand-bound receptors set off signaling cascades, which orient the protrusions the cells produce. Accordingly, directional migration is impaired in the absence of these receptors or when the relevant signaling pathways are inhibited (Sun and Devreotes, 1991; Doitsidou et al., 2002).

The precise molecular mechanisms that transform the activation of guidance receptors into blebbing at specific locations around the cell perimeter are not fully understood. In the case of GPCRs, receptor activation is associated with an elevation in the level of intracellular calcium that could, in turn, promote contractility at specific locations (Somlyo and Somlyo, 2003; Dhyani et al., 2020). Indeed, the increase in intracellular calcium is correlated with blebbing, and high calcium levels were observed in forming blebs (Blaser et al., 2006; Srivastava et al., 2020; Aoki et al., 2021). Consistently, knocking down Cxcr4b in zebrafish PGCs results in reduced calcium levels at the cell front. Conversely, an experimental increase in calcium levels at specific locations promotes the formation of blebs at those sites (Blaser et al., 2006). Another relevant signaling cascade activated by GPCRs is the Phosphoinositide 3-kinase (PI3K) pathway. Activation of guidance receptors results in activation of PI3K, which catalyzes the conversion of phosphatidylinositol (4,5)-bisphosphate (PIP2) to phosphatidylinositol 3,4,5 trisphosphate (PIP3) (Hemmings and Restuccia, 2012). Accordingly, higher levels of PIP3 are found at the cell front of neutrophils and *Dictyostelium,* where it is linked to increased actin polymerization and protrusion formation (Devreotes and Horwitz, 2015). In the context of blebbing, a reduction in the level of PIP3 was shown to reduce dynamic cell shape changes and protrusion formation (Dumstrei et al., 2004), and conversely, reduced levels of PIP2 are associated with increased blebbing (Zatulovskiy et al., 2014; Zhao et al., 2014; Bharadwaj et al., 2017). Since membrane linkers are activated by PIP2 binding (Fehon et al., 2010), the reduction in the level of this phospholipid could result in enhanced blebbing.

Importantly, cell polarization does not necessarily rely on external guidance cues, and protrusion formation at the front can be directed by cell-intrinsic self-guidance mechanisms [reviewed in (Wong and Gilmour, 2021)]. For example, in the absence of a chemoattractant, zebrafish PGCs, *Dictyostelium*, and cells using stable blebs can also polarize and migrate in random directions, indicating that bleb-based motility *per se* does not strictly require external guidance cues (Blaser et al., 2006; Yoshida and Soldati, 2006; Liu et al., 2015; Ruprecht et al., 2015; Olguin-Olguin et al., 2021).

**FIGURE 3:**
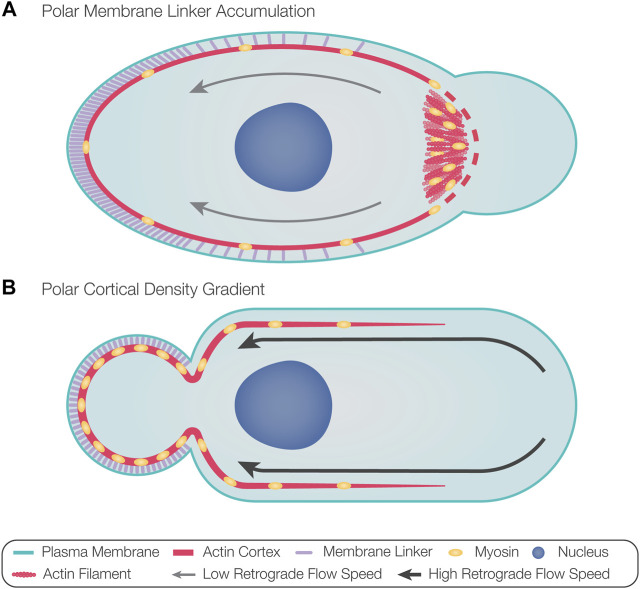
Models for directing bleb formation to the front of migrating cells. **(A)** Actin polymerization at the future leading edge provides a platform for the recruitment of myosin motors. Actomyosin polymerization and contraction result in actin retrograde flow, advecting membrane linker molecules toward the opposite aspect of the cell, thus defining the rear. Accumulation of membrane linker molecules at the rear prevents blebbing at this aspect of the cell. Increased actomyosin contractility at the front could introduce cortical breaks, which, in addition to the reduced levels of membrane linkers, could favor the formation of blebs at the leading edge. The polarized contractile activity could also result in local pressure elevation, favoring bleb formation if the hydrostatic pressure does not rapidly equilibrate throughout the cytosol. **(B)** Local fluctuations in contractility can cause the flow of actin and myosin toward the contractile region, thus dictating the position of the future rear. High retrograde flow speeds of polymerized actin from the front maintain polarization by establishing a cortical density gradient in the direction of the rear, thus favoring positioning of the bleb at the opposing side. Likewise, membrane linkers accumulate at the rear, potentially further inhibiting bleb formation at this aspect of the cell.

A recent study described the sequence of the intrinsic polarization cascade in zebrafish PGCs and how it can be biased by external cues (Olguin-Olguin et al., 2021). In this case, the first sign of polarization was found to be Rac1-driven enrichment of F-actin at the future leading edge of cells (Olguin-Olguin et al., 2021). This event was followed by the formation of blebs at this location and rearward actin retrograde flow that mediated the transport of linker molecules (e.g., ezrin) to the cell back (Olguin-Olguin et al., 2021). Indeed, using a photo-inducible version of Rac1, the position of the future leading edge could be experimentally directed (Olguin-Olguin et al., 2021). According to these findings, actin accumulation at the developing cell front provides a platform for myosin motors that induce contractility-dependent actin retrograde flow, which drives bleb-inhibiting proteins to the rear, thereby defining the front-back axis of the cell (Figure 3A). Here, the stochastic elevation of actin polymerization would initiate a polarization cascade in which the front and the back of the cell antagonize each other, thereby stabilizing the cell’s front-back axis. In this case, it was suggested that the sole function of the external guidance cue is to bias actin polymerization in the direction of the Cxcl12a chemokine source, thereby directing the migrating cell, which expresses the cognate receptor Cxcr4b, up the attractant gradient.

In motile cells migrating with stable blebs, fluctuations in cortical contractility were suggested to induce front-back polarization (Liu et al., 2015; Ruprecht et al., 2015). In this case, experimentally stimulating contractility dictated the position of the future rear, and the increase in cortical contractility initially resulted in a flow of actin and myosin toward the contractile region (Ruprecht et al., 2015). Polarization, then, was maintained by continuous retrograde actin flow, resulting in a cortical density gradient that increased in the direction of the rear (Figure 3B) (Liu et al., 2015; Ruprecht et al., 2015). Preservation of this actin retrograde flow requires constant rates of actin polymerization, depolymerization at the back of the cell, and diffusion of free actin and myosin toward the front (Liu et al., 2015; Ruprecht et al., 2015). Indeed, actin depolymerizing proteins ADF and cofilin-1 are found at the back of stable blebs and are required for sustaining retrograde actin flow (Ullo and Logue, 2021). This mode of polarization does not require external factors and can be initiated by intrinsic fluctuations in contractility.

The establishment of the cortical density gradient in stable bleb migration is correlated with the high rates of retrograde actin flow observed in these cells. Specifically, in such cells, retrograde actin flow speeds of 15.8 μm/min in central parts of the cell and up to 150 μm/min at the cell front were measured (Liu et al., 2015; Ruprecht et al., 2015). In comparison, intermittently blebbing zebrafish PGCs show retrograde actin flow speeds of up to 1.7 μm/min (Grimaldi et al., 2020). The very high speed of the retrograde flow in stable bleb migrating cells could account for the observation that actin and myosin are found primarily at the back of the cells, in contrast with the presence of actin and myosin at the front of intermittently blebbing cells. The significance of retrograde flow speeds for the differences between stable bleb-forming and intermittently blebbing cells in controlling the subcellular localization of actin was demonstrated in zebrafish PGCs, where an experimental enhancement of retrograde flow speed to about 7 μm/min resulted in increased accumulation of actin at the rear (Kardash et al., 2010; Grimaldi et al., 2020).

In summary, the distribution of membrane-cortex linkers, actin polymerization, and contractility were shown to direct bleb formation to the leading edge in guided and non-guided cells.

## Factors Promoting Bleb Formation

**FIGURE 4:**
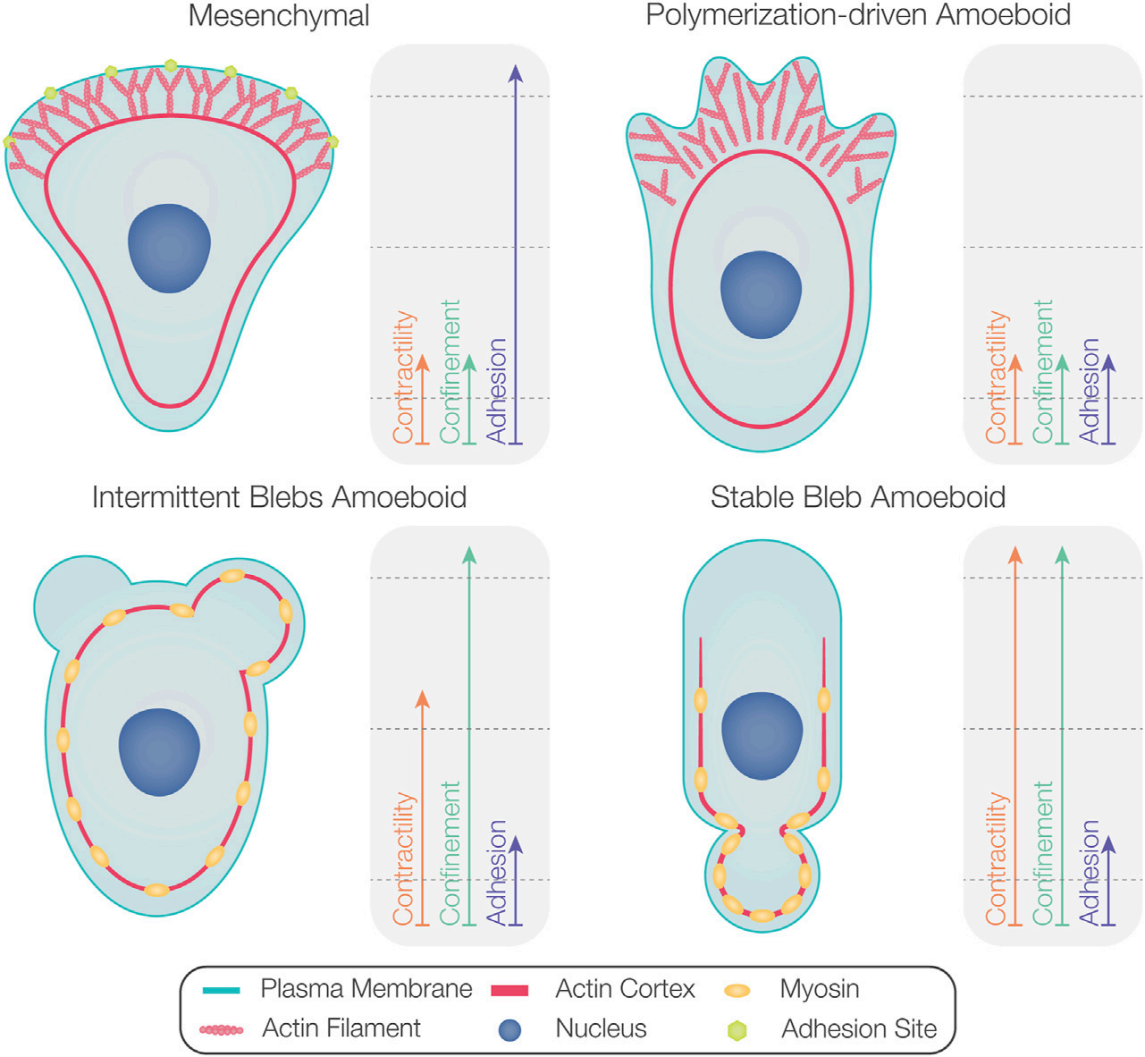
Factors determining the degree of bleb formation in migrating cells. While mesenchymal motility is associated with high levels of substrate adhesion (upper left), polymerization-driven amoeboid motility occurs at lower levels of substrate adhesion (upper right). As the contractility level increases, amoeboid-motile cells form more blebs than polymerization-driven protrusions (bottom left). Cells displaying non-persistent bleb-based motility can switch to stable bleb migration at very high contractility levels (bottom right). Confinement can also promote blebbing activity.

Certain cell types primarily produce blebs during their migration (e.g., zebrafish PGCs (Blaser et al., 2006), *Fundulus heteroclitus* deep cells (Fink and Trinkaus, 1988), and certain tumor cells (Sahai and Marshall, 2003). Interestingly, amoeboid-motile cells such as *Dictyostelium* (Yoshida and Soldati, 2006; Zatulovskiy et al., 2014), zebrafish prechordal plate progenitors (Diz-Muñoz et al., 2010), and late blastulae *Fundulus* deep cells (Trinkaus, 1973) form both blebs and polymerization-driven protrusions. In these cases, certain environmental conditions and changes in intracellular activities facilitate shifts from one protrusion formation mode to another. In the following subsections, we present factors that influence the proportion of blebs versus polymerization-driven protrusions (summarized in Figure 4).

The factors we discuss in the subsections below are relevant for pathological conditions as well. A key feature characterizing neoplastic diseases is activation of cell invasion and metastasis, processes where bleb formation is observed. Certain cancer cell lines (e.g., M2 melanoma cells, Walker cells, human fibrosarcoma HT1080 cells, and MDA-MB-231 breast cancer cells) were shown to form blebs (Cunningham et al., 1992; Keller and Bebie, 1996; Friedl and Wolf, 2003), and under confinement cells such as Walker cells, A375 melanoma cells, A549 human lung cancer cells, and U2OS human osteosarcoma cells display stable bleb migration (Bergert et al., 2015; Logue et al., 2015). The ability to switch between protrusion types is thought to increase the plasticity of metastatic cancer cells by allowing them to adapt to features in the environment (Friedl, 2004; Friedl and Wolf, 2010; Paňková et al., 2010; Sanz-Moreno and Marshall, 2010; Taddei et al., 2013).

### Actomyosin Contractility and Actin Polymerization

High actomyosin contractility and increased cortical tension are the hallmarks of motile cells producing blebs (Langridge and Kay, 2006; Yoshida and Soldati, 2006; Bergert et al., 2012; Zatulovskiy et al., 2014). Indeed, migrating cells can be directed to generate more blebs by increasing contractility. For example, zebrafish mesodermal progenitor cells that primarily display polymerization-driven protrusions form more blebs upon myosin activation (Ruprecht et al., 2015). Similarly, expression of a constitutively active form of ROCK in Walker cells reduces the formation of polymerization-driven protrusions with a concomitant increase in blebbing activity (Bergert et al., 2012). Conversely, *Dictyostelium* and leukocytes develop predominantly polymerization-driven protrusions when myosin function is inhibited (Langridge and Kay, 2006; Yoshida and Soldati, 2006; Lämmermann et al., 2008; Jacobelli et al., 2009). Thus, in the context of amoeboid migration, the level of contractility is a major factor that dictates the protrusion type cells produce, with the stable bleb phenomenon representing an extreme case of high level of myosin activation (Liu et al., 2015; Ruprecht et al., 2015). In support of this, as mentioned above, non-manipulated migrating zebrafish PGCs form intermittent blebs, but the expression of constitutively active RhoA protein results in the formation of a large stable bleb (Kardash et al., 2010; Grimaldi et al., 2020; Olguin-Olguin et al., 2021). Consistently, lowering contractility in cells forming stable blebs leads to intermittent protrusion formation (Ruprecht et al., 2015).

Similar to non-transformed cells, bleb-based motility of cancer cells within 3D environments was shown to require Rho/ROCK signaling (Sahai and Marshall, 2003). Moreover, increased contractility promotes the invasiveness of mouse embryonic fibroblasts and Chinese hamster ovary (CHO) cells (Gadea et al., 2007; Tournaviti et al., 2007). Since factors associated with bleb formation, particularly those involved in regulating contractility, show elevated activity in cancer cells, they constitute attractive targets for inhibiting metastasis.

Experimental evidence suggests that factors promoting actin polymerization-driven protrusion and those driving blebs act antagonistically. For example, impairing the activity of Arp2/3 reduces actin polymerization and increases blebbing in Walker cells (Bergert et al., 2012), in melanoma cells (Logue et al., 2018), in *Dictyostelium* (Langridge and Kay, 2006; Zatulovskiy et al., 2014), as well as in embryonic *Caenorhabditis elegans* cells (Severson et al., 2002; Roh-Johnson and Goldstein, 2009). Conversely, loss of function of coronin and profilins that are important for actin assembly and stabilization leads to a reduction in blebbing in *Dictyostelium* (Zatulovskiy et al., 2014). The same effect can be achieved by stimulating actin polymerization *via* jasplakinolide treatment in cultured cell lines (Laser-Azogui et al., 2014). Similarly, upon increasing Rac1 activity, Walker cells show a reduction of blebs and a concomitant increase in polymerization-driven protrusions, presumably stemming from alterations in cortex composition and cortical tension (Bergert et al., 2012).

### Confinement

Generally, blebs are associated with the movement of cells within 3D environments. For example, zebrafish PGCs (Blaser et al., 2006), other cell types that migrate within early embryos (Fink and Trinkaus, 1988; Diz-Muñoz et al., 2010), and tumor cells form blebs as they migrate within 3D matrices (Sahai and Marshall, 2003; Wolf et al., 2003). Interestingly, when *Dictyostelium* cells undergo transition to multicellular development, they show an increase in bleb formation (Zatulovskiy et al., 2014). These observations led to the hypothesis that confined environments could promote the formation of blebs.

Indeed, experimentally confining *Dictyostelium* cells using agarose gel overlays (Zatulovskiy et al., 2014) or microfluidic confinement chambers (Ibo et al., 2016) leads to an increase in blebbing. Similarly, the mechanical load exerted by uniaxial compression is sufficient to induce blebbing in *Dictyostelium* (Srivastava et al., 2017), and the extent of mechanical load positively scales with the number of observed blebs relative to polymerization-driven protrusions (Srivastava et al., 2020). This phenomenon was also observed in multicellular organisms, such as in the case of zebrafish mesodermal progenitor cells that show an increased size of blebs upon confinement (Ruprecht et al., 2015). Additionally, the rigidity of the confining substrate scales with the number of blebs formed in *Dictyostelium* cells, where increasing the mechanical resistance of confining agarose overlays increases the number of blebs (Zatulovskiy et al., 2014). The effect of confinement on protrusion types was also observed in several types of mammalian cells in which confinement increases the formation of blebs (Liu et al., 2015).

Mechanistically, confinement was shown to be sensed, for instance, by the stretch-operated Piezo channels and deformation of the nucleus (Lomakin et al., 2020; Srivastava et al., 2020; Venturini et al., 2020). The actual response to confinement is considered to reflect an increase in actomyosin contractility, as observed in confined zebrafish mesodermal progenitor cells and *Dictyostelium* cells, which show an elevated cortical accumulation of myosin under these conditions (Ruprecht et al., 2015; Srivastava et al., 2020). The functional importance of enhanced contractility for migration of blebbing cells under confined conditions was demonstrated in *Dictyostelium,* where inhibiting contractility led to reduced protrusion formation and lower migration speed (Zatulovskiy et al., 2014).

### Adhesion

Adhesion is an additional important parameter that influences the type of protrusions cells form. Migrating cells need to transmit forces to their surroundings, which allows them to advance forward. A hallmark of mesenchymal migration is tight adhesion to extracellular matrix components *via* structures called focal adhesions that contain transmembrane integrin molecules. In this case, following the adhesion of the protrusion to the ECM, the cells pull themselves forward (Ananthakrishnan and Ehrlicher, 2007). However, cells have also been shown to migrate without integrins and, thus, without specific interactions with the substratum (Lämmermann et al., 2008; Paluch et al., 2015). In general, amoeboid migration occurs under low adhesion conditions, and zebrafish mesodermal progenitor cells that migrate using polymerization-driven protrusions lose their ability to migrate on adhesive 2D substrates when induced to form blebs (Ruprecht et al., 2015). The inability to migrate on 2D substrates was also reported for blebbing Walker cells (Bergert et al., 2012).

In addition to these observations, reduced cell adhesion or reduced formation of focal adhesions have been shown to be instructive regarding the switch from mesenchymal to amoeboid motility (Liu et al., 2015), and disrupting focal adhesion formation was reported to result in an increase in blebbing (Logue et al., 2018). Consistently, blebbing of Walker cells is inhibited by confining them under adhesive substrate (Bergert et al., 2012). Thus, low adhesion is a common feature of amoeboid migration.

## The Role of Blebs in Migration

In the case of mesenchymal cell migration, cells utilize retrograde flow of actin in the lamellipodia coupled with focal adhesions to generate traction against the substrate (Case and Waterman, 2015; Elosegui-Artola et al., 2018). In contrast, although blebs are common and, in some cases, the only type of protrusions migrating amoeboid cells form, their actual contribution to locomotion is not fully understood. Several models have been formulated to explain how amoeboid motile cells could generate traction in an adhesion-independent manner [reviewed in (Paluch et al., 2015)]. For instance, amoeboid cells could connect to their environment by exerting lateral pushing forces that, coupled with the extension of protrusions at the leading edge, could result in cell body translocation (Charras and Paluch, 2008; Renkawitz and Sixt, 2010; Paluch and Raz, 2013). In addition, it has been proposed that retrograde flow provides friction *via* nonspecific interactions with the substrate (Hawkins et al., 2011; Ruprecht et al., 2015). Such friction could be generated by any cell-surface molecule that directly or indirectly interacts with actin filaments undergoing retrograde flow (Paluch et al., 2015). However, both motility *via* lateral pushing and nonspecific friction do not necessarily require expansion of blebs for movement. Therefore, an interesting open question is whether blebs contribute to amoeboid motility or whether they represent an epiphenomenon of the increased contractility required for this type of migration.

Related to this question, a mathematical model investigating the significance of protrusion types suggested that blebs could be beneficial in specific extracellular environments (Tozluoğlu et al., 2013). This work demonstrated that both polymerization-driven protrusions and blebs could promote locomotion in continuous confined environments (modeling cells squeezed between planar sheets). Importantly, this study suggests that in discontinuous confining environments (analogous to a collagen mesh), bleb-based migration is more effective in generating traction and translocation of the cell forward due to more effective intercalation into gaps (Tozluoğlu et al., 2013). Indeed, it was shown that migrating Schwann cells form lateral blebs, which could assist migration by intercalating within protrusions of adjacent cells (Cattin et al., 2015). While this would be an interesting use of blebs in cell motility, direct experimental support for this mechanism is still lacking.

In addition to the mechanistic involvement of blebs in locomotion, blebs could also contribute to controlling migration precision. Motile zebrafish mesendodermal progenitor cells display phases in which they primarily form blebs and phases in which polymerization-driven protrusions are predominant (Diz-Muñoz et al., 2016). Modulating the time that cells spend in either of these phases was shown to influence migration precision (Diz-Muñoz et al., 2016). Furthermore, stable bleb migration was suggested to allow fast extrusion of cells from contractile embryonic regions (Ruprecht et al., 2015).

Last, while mesenchymal migration allows specific interactions of cells with other cells or substrates, allowing processes such as haptotaxis (directional migration upward an adhesion gradient) and eventual formation of stable connections, blebs were suggested to be preferred when long-lasting interactions are less important, as is the case in chemotaxis (Charras and Sahai, 2014). Indeed, certain cells specialized for chemotaxis produce blebs when exposed to chemoattractants (Blaser et al., 2006; Langridge and Kay, 2006), and in *Dictyostelium*, the steepness of the chemoattractant gradient was shown to increase bleb formation (Ibo et al., 2016).

Taken together, cells form different types of protrusions based on external cues, as well as the expression of migration-relevant components within them. The advantages of migrating by employing blebs or polymerization-driven protrusions are most likely specific to the environment within which the cells are located. Determining the significance of protrusion types for migration *in vivo* would require detailed quantitative analysis of motility parameters of cell types that can form blebs and polymerization-driven protrusions. Accordingly, it should be determined whether these protrusion types provide an advantage in specific contexts.

## Conclusion

Blebs are considered to be a major type of protrusion in the context of amoeboid cell migration. Multiple mechanisms regulate the formation of blebs and experimental data points at components and activities that bias the formation of blebs to the leading edge of the migrating cell. Whereas the polar distribution of membrane-cortex linkers has been shown to control the position of bleb formation, the significance of the subcellular localization of myosin motors and the relevance of specific signaling pathways are not as well defined.

Although blebs are a prevalent protrusion type in migrating cells, and the mechanisms promoting their formation have been to a large extent explored, determining their precise mechanistic functions in promoting cell motility in 3D environments are not as clear. In particular, it is not known whether blebs are strictly required for migration under specific conditions and if they provide an advantage over other protrusion strategies in these contexts. Given the ability of cells to dynamically modify the types of protrusions they produce, it is possible that blebs and polymerization-driven protrusions represent extremes within a spectrum. Accordingly, the precise features of protrusions and their abundance are influenced by environmental conditions and can be controlled by the extent of actin polymerization and actomyosin contraction.

## References

[B1] Abercrombie M. Joan E. Heaysman M. Pegrum S. M. (1970). The Locomotion of Fibroblasts in Culture. Exp. Cell Res. 60, 437–444. 10.1016/0014-4827(70)90537-9 5463639

[B2] Ananthakrishnan R. Ehrlicher A. (2007). The Forces behind Cell Movement. Int. J. Biol. Sci. 3, 303–317. 10.7150/ijbs.3.303 17589565 PMC1893118

[B3] Aoki K. Harada S. Kawaji K. Matsuzawa K. Uchida S. Ikenouchi J. (2021). STIM-Orai1 Signaling Regulates Fluidity of Cytoplasm during Membrane Blebbing. Nat. Commun. 12, 480. 10.1038/s41467-020-20826-5 33473127 PMC7817837

[B4] Babich A. Li S. O'Connor R. S. Milone M. C. Freedman B. D. Burkhardt J. K. (2012). F-Actin Polymerization and Retrograde Flow Drive Sustained PLCγ1 Signaling during T Cell Activation. J. Cell Biol. 197, 775–787. 10.1083/jcb.201201018 22665519 PMC3373411

[B5] Bergert M. Chandradoss S. D. Desai R. A. Paluch E. (2012). Cell Mechanics Control Rapid Transitions between Blebs and Lamellipodia during Migration. Proc. Natl. Acad. Sci. U.S.A. 109, 14434–14439. 10.1073/pnas.1207968109 22786929 PMC3437886

[B6] Bergert M. Erzberger A. Desai R. A. Aspalter I. M. Oates A. C. Charras G. (2015). Force Transmission during Adhesion-independent Migration. Nat. Cell Biol. 17, 524–529. 10.1038/ncb3134 25774834 PMC6485532

[B7] Bharadwaj R. Arya R. Shahid mansuri M. Bhattacharya S. Bhattacharya A. (2017). EhRho1 Regulates Plasma Membrane Blebbing through PI3 Kinase inEntamoeba Histolytica. Cell. Microbiol. 19, e12751. 10.1111/cmi.12751 28477431

[B8] Blaser H. Reichman-Fried M. Castanon I. Dumstrei K. Marlow F. L. Kawakami K. (2006). Migration of Zebrafish Primordial Germ Cells: A Role for Myosin Contraction and Cytoplasmic Flow. Dev. Cell 11, 613–627. 10.1016/j.devcel.2006.09.023 17084355

[B9] Boldajipour B. Mahabaleshwar H. Kardash E. Reichman-Fried M. Blaser H. Minina S. (2008). Control of Chemokine-Guided Cell Migration by Ligand Sequestration. Cell 132, 463–473. 10.1016/j.cell.2007.12.034 18267076

[B10] Case L. B. Waterman C. M. (2015). Integration of Actin Dynamics and Cell Adhesion by a Three-Dimensional, Mechanosensitive Molecular Clutch. Nat. Cell Biol. 17, 955–963. 10.1038/ncb3191 26121555 PMC6300998

[B11] Cattin A.-L. Burden J. J. Van Emmenis L. Mackenzie F. E. Hoving J. J. A. Garcia Calavia N. (2015). Macrophage-Induced Blood Vessels Guide Schwann Cell-Mediated Regeneration of Peripheral Nerves. Cell 162, 1127–1139. 10.1016/j.cell.2015.07.021 26279190 PMC4553238

[B12] Charras G. Paluch E. (2008). Blebs Lead the Way: How to Migrate without Lamellipodia. Nat. Rev. Mol. Cell Biol. 9, 730–736. 10.1038/nrm2453 18628785

[B13] Charras G. Sahai E. (2014). Physical Influences of the Extracellular Environment on Cell Migration. Nat. Rev. Mol. Cell Biol. 15, 813–824. 10.1038/nrm3897 25355506

[B14] Charras G. T. Coughlin M. Mitchison T. J. Mahadevan L. (2008). Life and Times of a Cellular Bleb. Biophysical J. 94, 1836–1853. 10.1529/biophysj.107.113605 PMC2242777 17921219

[B15] Charras G. T. Hu C.-K. Coughlin M. Mitchison T. J. (2006). Reassembly of Contractile Actin Cortex in Cell Blebs. J. Cell Biol. 175, 477–490. 10.1083/jcb.200602085 17088428 PMC2064524

[B16] Charras G. T. Mitchison T. J. Mahadevan L. (2009). Animal Cell Hydraulics. J. Cell Sci. 122, 3233–3241. 10.1242/jcs.049262 19690051 PMC2736862

[B17] Charras G. T. Yarrow J. C. Horton M. A. Mahadevan L. Mitchison T. J. (2005). Non-equilibration of Hydrostatic Pressure in Blebbing Cells. Nature 435, 365–369. 10.1038/nature03550 15902261 PMC1564437

[B18] Collier S. Paschke P. Kay R. R. Bretschneider T. (2017). Image Based Modeling of Bleb Site Selection. Sci. Rep. 7, 6692. 10.1038/s41598-017-06875-9 28751725 PMC5532237

[B19] Cunningham C. C. (1995). Actin Polymerization and Intracellular Solvent Flow in Cell Surface Blebbing. J. Cell Biol. 129, 1589–1599. 10.1083/jcb.129.6.1589 7790356 PMC2291187

[B20] Cunningham C. C. Gorlin J. B. Kwiatkowski D. J. Hartwig J. H. Janmey P. A. Byers H. R. (1992). Actin-Binding Protein Requirement for Cortical Stability and Efficient Locomotion. Science 255, 325–327. 10.1126/science.1549777 1549777

[B21] Devreotes P. Horwitz A. R. (2015). Signaling Networks that Regulate Cell Migration. Cold Spring Harb. Perspect. Biol. 7, a005959. 10.1101/cshperspect.a005959 26238352 PMC4526752

[B22] Dhyani V. Gare S. Gupta R. K. Swain S. Venkatesh K. V. Giri L. (2020). GPCR Mediated Control of Calcium Dynamics: A Systems Perspective. Cell. Signal. 74, 109717. 10.1016/j.cellsig.2020.109717 32711109 PMC7375278

[B23] Diz-Muñoz A. Krieg M. Bergert M. Ibarlucea-Benitez I. Muller D. J. Paluch E. (2010). Control of Directed Cell Migration *In Vivo* by Membrane-To-Cortex Attachment. Plos Biol. 8, e1000544. 10.1371/journal.pbio.1000544 21151339 PMC2994655

[B24] Diz-Muñoz A. Romanczuk P. Yu W. Bergert M. Ivanovitch K. Salbreux G. (2016). Steering Cell Migration by Alternating Blebs and Actin-Rich Protrusions. Bmc Biol. 14, 74. 10.1186/s12915-016-0294-x 27589901 PMC5010735

[B25] Doitsidou M. Reichman-Fried M. Stebler J. Köprunner M. Dörries J. Meyer D. (2002). Guidance of Primordial Germ Cell Migration by the Chemokine SDF-1. Cell 111, 647–659. 10.1016/s0092-8674(02)01135-2 12464177

[B26] Dumstrei K. Mennecke R. Raz E. (2004). Signaling Pathways Controlling Primordial Germ Cell Migration in Zebrafish. J. Cell Sci. 117, 4787–4795. 10.1242/jcs.01362 15340012

[B27] Elosegui-Artola A. Trepat X. Roca-Cusachs P. (2018). Control of Mechanotransduction by Molecular Clutch Dynamics. Trends Cell Biol. 28, 356–367. 10.1016/j.tcb.2018.01.008 29496292

[B28] Fackler O. T. Grosse R. (2008). Cell Motility through Plasma Membrane Blebbing. J. Cell Biol. 181, 879–884. 10.1083/jcb.200802081 18541702 PMC2426937

[B29] Fehon R. G. McClatchey A. I. Bretscher A. (2010). Organizing the Cell Cortex: the Role of ERM Proteins. Nat. Rev. Mol. Cell Biol. 11, 276–287. 10.1038/nrm2866 20308985 PMC2871950

[B30] Fink R. D. Trinkaus J. P. (1988). Fundulus Deep Cells: Directional Migration in Response to Epithelial Wounding. Dev. Biol. 129, 179–190. 10.1016/0012-1606(88)90172-8 3410158

[B31] Friedl P. (2004). Prespecification and Plasticity: Shifting Mechanisms of Cell Migration. Curr. Opin. Cell Biol. 16, 14–23. 10.1016/j.ceb.2003.11.001 15037300

[B32] Friedl P. Wolf K. (2010). Plasticity of Cell Migration: a Multiscale Tuning Model. J. Cell Biol. 188, 11–19. 10.1083/jcb.200909003 19951899 PMC2812848

[B33] Friedl P. Wolf K. (2003). Tumour-cell Invasion and Migration: Diversity and Escape Mechanisms. Nat. Rev. Cancer 3, 362–374. 10.1038/nrc1075 12724734

[B34] Fritz-Laylin L. K. Lord S. J. Kakley M. Mullins R. D. (2018). Concise Language Promotes Clear Thinking about Cell Shape and Locomotion. Bioessays 40, 1700225. 10.1002/bies.201700225 PMC6175535 29846958

[B35] Gabbireddy S. R. Vosatka K. W. Chung A. J. Logue J. S. (2021). Melanoma Cells Adopt Features of Both Mesenchymal and Amoeboid Migration within Confining Channels. Sci. Rep. 11, 17804. 10.1038/s41598-021-97348-7 34493759 PMC8423822

[B36] Gadea G. de Toledo M. Anguille C. Roux P. (2007). Loss of P53 Promotes RhoA-ROCK-dependent Cell Migration and Invasion in 3D Matrices. J. Cell Biol. 178, 23–30. 10.1083/jcb.200701120 17606864 PMC2064414

[B37] Goudarzi M. Banisch T. U. Mobin M. B. Maghelli N. Tarbashevich K. Strate I. (2012). Identification and Regulation of a Molecular Module for Bleb-Based Cell Motility. Dev. Cell 23, 210–218. 10.1016/j.devcel.2012.05.007 22705393

[B38] Goudarzi M. Boquet-Pujadas A. Olivo-Marin J.-C. Raz E. (2019). Fluid Dynamics during Bleb Formation in Migrating Cells *In Vivo* . Plos One 14, e0212699. 10.1371/journal.pone.0212699 30807602 PMC6391022

[B39] Goudarzi M. Tarbashevich K. Mildner K. Begemann I. Garcia J. Paksa A. (2017). Bleb Expansion in Migrating Cells Depends on Supply of Membrane from Cell Surface Invaginations. Dev. Cell 43, 577–587. e5. 10.1016/j.devcel.2017.10.030 29173819 PMC5939956

[B40] Grimaldi C. Raz E. (2020). Germ Cell Migration-Evolutionary Issues and Current Understanding. Seminars Cell & Dev. Biol. 100, 152–159. 10.1016/j.semcdb.2019.11.015 31864795

[B41] Grimaldi C. Schumacher I. Boquet-Pujadas A. Tarbashevich K. Vos B. E. Bandemer J. (2020). E-Cadherin Focuses Protrusion Formation at the Front of Migrating Cells by Impeding Actin Flow. Nat. Commun. 11, 5397. 10.1038/s41467-020-19114-z 33106478 PMC7588466

[B42] Gutjahr M. C. Rossy J. Niggli V. (2005). Role of Rho, Rac, and Rho-Kinase in Phosphorylation of Myosin Light Chain, Development of Polarity, and Spontaneous Migration of Walker 256 Carcinosarcoma Cells. Exp. Cell Res. 308, 422–438. 10.1016/j.yexcr.2005.05.001 15950966

[B43] Hawkins R. J. Poincloux R. Bénichou O. Piel M. Chavrier P. Voituriez R. (2011). Spontaneous Contractility-Mediated Cortical Flow Generates Cell Migration in Three-Dimensional Environments. Biophysical J. 101, 1041–1045. 10.1016/j.bpj.2011.07.038 PMC3164128 21889440

[B44] Heid P. J. Geiger J. Wessels D. Voss E. Soll D. R. (2005). Computer-assisted Analysis of Filopod Formation and the Role of Myosin II Heavy Chain Phosphorylation inDictyostelium. J. Cell Sci. 118, 2225–2237. 10.1242/jcs.02342 15855234

[B45] Hemmings B. A. Restuccia D. F. (2012). PI3K-PKB/Akt Pathway. Cold Spring Harb. Perspect. Biol. 4, a011189. 10.1101/cshperspect.a011189 22952397 PMC3428770

[B46] Henson J. H. Svitkina T. M. Burns A. R. Hughes H. E. MacPartland K. J. Nazarian R. (1999). Two Components of Actin-Based Retrograde Flow in Sea Urchin Coelomocytes. MBoC 10, 4075–4090. 10.1091/mbc.10.12.4075 10588644 PMC25744

[B47] Hind L. E. Vincent W. J. B. Huttenlocher A. (2016). Leading from the Back: The Role of the Uropod in Neutrophil Polarization and Migration. Dev. Cell 38, 161–169. 10.1016/j.devcel.2016.06.031 27459068 PMC4982870

[B48] Ibo M. Srivastava V. Robinson D. N. Gagnon Z. R. (2016). Cell Blebbing in Confined Microfluidic Environments. Plos One 11, e0163866. 10.1371/journal.pone.0163866 27706201 PMC5051935

[B49] Ikenouchi J. Aoki K. (2021). A Clockwork Bleb: Cytoskeleton, Calcium, and Cytoplasmic Fluidity. Febs J. 10.1111/febs.16220 34614290

[B50] Insall R. H. Soede R. D. Schaap P. Devreotes P. N. (1994). Two cAMP Receptors Activate Common Signaling Pathways in Dictyostelium. MBoC 5, 703–711. 10.1091/mbc.5.6.703 7949426 PMC301085

[B51] Jacobelli J. Bennett F. C. Pandurangi P. Tooley A. J. Krummel M. F. (2009). Myosin-IIA and ICAM-1 Regulate the Interchange between Two Distinct Modes of T Cell Migration. J. Immunol. 182, 2041–2050. 10.4049/jimmunol.0803267 19201857

[B52] Kardash E. Reichman-Fried M. Maître J.-L. Boldajipour B. Papusheva E. Messerschmidt E.-M. (2010). A Role for Rho GTPases and Cell-Cell Adhesion in Single-Cell Motility *In Vivo* . Nat. Cell Biol. 12, 47–53. 10.1038/ncb2003 20010816

[B53] Keller H. U. Bebie H. (1996). Protrusive Activity Quantitatively Determines the Rate and Direction of Cell Locomotion. Cell Motil. Cytoskelet. 33, 241–251. 10.1002/(sici)1097-0169(1996)33:4<241::aid-cm1>3.0.co;2-c 8801030

[B54] Lämmermann T. Bader B. L. Monkley S. J. Worbs T. Wedlich-Söldner R. Hirsch K. (2008). Rapid Leukocyte Migration by Integrin-independent Flowing and Squeezing. Nature 453, 51–55. 10.1038/nature06887 18451854

[B55] Lämmermann T. Sixt M. (2009). Mechanical Modes of 'amoeboid' Cell Migration. Curr. Opin. Cell Biol. 21, 636–644. 10.1016/j.ceb.2009.05.003 19523798

[B56] Langridge P. D. Kay R. R. (2006). Blebbing of Dictyostelium Cells in Response to Chemoattractant. Exp. Cell Res. 312, 2009–2017. 10.1016/j.yexcr.2006.03.007 16624291

[B57] Laser-Azogui A. Diamant-Levi T. Israeli S. Roytman Y. Tsarfaty I. (2014). Met-induced Membrane Blebbing Leads to Amoeboid Cell Motility and Invasion. Oncogene 33, 1788–1798. 10.1038/onc.2013.138 23665680

[B58] Laster S. M. Mackenzie J. M. (1996). Bleb Formation and F-Actin Distribution during Mitosis and Tumor Necrosis Factor-Induced Apoptosis. Microsc. Res. Tech. 34, 272–280. 10.1002/(sici)1097-0029(19960615)34:3<272::aid-jemt10>3.0.co;2-j 8743415

[B59] Liu Y.-J. Le Berre M. Lautenschlaeger F. Maiuri P. Callan-Jones A. Heuzé M. (2015). Confinement and Low Adhesion Induce Fast Amoeboid Migration of Slow Mesenchymal Cells. Cell 160, 659–672. 10.1016/j.cell.2015.01.007 25679760

[B60] Logue J. S. Cartagena-Rivera A. X. Baird M. A. Davidson M. W. Chadwick R. S. Waterman C. M. (2015). Erk Regulation of Actin Capping and Bundling by Eps8 Promotes Cortex Tension and Leader Bleb-Based Migration. Elife 4, e08314. 10.7554/elife.08314 26163656 PMC4522647

[B61] Logue J. S. Cartagena-Rivera A. X. Chadwick R. S. (2018). c-Src Activity Is Differentially Required by Cancer Cell Motility Modes. Oncogene 37, 2104–2121. 10.1038/s41388-017-0071-5 29379163 PMC5906457

[B62] Lomakin A. J. Cattin C. J. Cuvelier D. Alraies Z. Molina M. Nader G. P. F. (2020). The Nucleus Acts as a Ruler Tailoring Cell Responses to Spatial Constraints. Science 370, eaba2894. 10.1126/science.aba2894 33060332 PMC8059074

[B63] Lorentzen A. Bamber J. Sadok A. Elson-Schwab I. Marshall C. J. (2011). An Ezrin-Rich, Rigid Uropod-like Structure Directs Movement of Amoeboid Blebbing Cells. J. Cell Sci. 124, 1256–1267. 10.1242/jcs.074849 21444753

[B64] Maugis B. Brugués J. Nassoy P. Guillen N. Sens P. Amblard F. (2010). Dynamic Instability of the Intracellular Pressure Drives Bleb-Based Motility. J. Cell Sci. 123, 3884–3892. 10.1242/jcs.065672 20980385

[B65] Meyen D. Tarbashevich K. Banisch T. U. Wittwer C. Reichman-Fried M. Maugis B. (2015). Dynamic Filopodia Are Required for Chemokine-dependent Intracellular Polarization during Guided Cell Migration *In Vivo* . Elife 4, e05279. 10.7554/elife.05279 PMC4397908 25875301

[B66] Mitchison T. J. Charras G. T. Mahadevan L. (2008). Implications of a Poroelastic Cytoplasm for the Dynamics of Animal Cell Shape. Seminars Cell & Dev. Biol. 19, 215–223. 10.1016/j.semcdb.2008.01.008 PMC2696638 18395478

[B67] Mitchison T. Kirschner M. (1988). Cytoskeletal Dynamics and Nerve Growth. Neuron 1, 761–772. 10.1016/0896-6273(88)90124-9 3078414

[B68] Olguin-Olguin A. Aalto A. Maugis B. Boquet-Pujadas A. Hoffmann D. Ermlich L. (2021). Chemokine-biased Robust Self-Organizing Polarization of Migrating Cells *In Vivo* . Proc. Natl. Acad. Sci. U.S.A. 118, e2018480118. 10.1073/pnas.2018480118 33574063 PMC7896345

[B69] Paluch E. K. Aspalter I. M. Sixt M. (2016). Focal Adhesion-independent Cell Migration. Annu. Rev. Cell Dev. Biol. 32, 469–490. 10.1146/annurev-cellbio-111315-125341 27501447

[B70] Paluch E. K. Raz E. (2013). The Role and Regulation of Blebs in Cell Migration. Curr. Opin. Cell Biol. 25, 582–590. 10.1016/j.ceb.2013.05.005 23786923 PMC3989058

[B71] Paluch E. Piel M. Prost J. Bornens M. Sykes C. (2005). Cortical Actomyosin Breakage Triggers Shape Oscillations in Cells and Cell Fragments. Biophysical J. 89, 724–733. 10.1529/biophysj.105.060590 PMC1366569 15879479

[B72] Paňková K. Rösel D. Novotný M. Brábek J. (2010). The Molecular Mechanisms of Transition between Mesenchymal and Amoeboid Invasiveness in Tumor Cells. Cell. Mol. Life Sci. 67, 63–71. 10.1007/s00018-009-0132-1 19707854 PMC2801846

[B73] Pinner S. Sahai E. (2008). PDK1 Regulates Cancer Cell Motility by Antagonising Inhibition of ROCK1 by RhoE. Nat. Cell Biol. 10, 127–137. 10.1038/ncb1675 18204440

[B74] Pollard T. D. Blanchoin L. Mullins R. D. (2000). Molecular Mechanisms Controlling Actin Filament Dynamics in Nonmuscle Cells. Annu. Rev. Biophys. Biomol. Struct. 29, 545–576. 10.1146/annurev.biophys.29.1.545 10940259

[B75] Raz E. (2004). Guidance of Primordial Germ Cell Migration. Curr. Opin. Cell Biol. 16, 169–173. 10.1016/j.ceb.2004.01.004 15196560

[B76] Renkawitz J. Sixt M. (2010). Mechanisms of Force Generation and Force Transmission during Interstitial Leukocyte Migration. Embo Rep. 11, 744–750. 10.1038/embor.2010.147 20865016 PMC2948197

[B77] Ridley A. J. (2015). Rho GTPase Signalling in Cell Migration. Curr. Opin. Cell Biol. 36, 103–112. 10.1016/j.ceb.2015.08.005 26363959 PMC4728192

[B78] Ridley A. J. (2001). Rho GTPases and Cell Migration. J. Cell Sci. 114, 2713–2722. 10.1242/jcs.114.15.2713 11683406

[B79] Roh-Johnson M. Goldstein B. (2009). *In Vivo* roles for Arp2/3 in Cortical Actin Organization during *C. elegans* Gastrulation. J. Cell Sci. 122, 3983–3993. 10.1242/jcs.057562 19889970 PMC2773197

[B80] Rossy J. Gutjahr M. C. Blaser N. Schlicht D. Niggli V. (2007). Ezrin/moesin in Motile Walker 256 Carcinosarcoma Cells: Signal-dependent Relocalization and Role in Migration. Exp. Cell Res. 313, 1106–1120. 10.1016/j.yexcr.2006.12.023 17292355

[B81] Rottner K. Schaks M. (2019). Assembling Actin Filaments for Protrusion. Curr. Opin. Cell Biol. 56, 53–63. 10.1016/j.ceb.2018.09.004 30278304

[B82] Ruprecht V. Wieser S. Callan-Jones A. Smutny M. Morita H. Sako K. (2015). Cortical Contractility Triggers a Stochastic Switch to Fast Amoeboid Cell Motility. Cell 160, 673–685. 10.1016/j.cell.2015.01.008 25679761 PMC4328143

[B83] Sahai E. Marshall C. J. (2003). Differing Modes of Tumour Cell Invasion Have Distinct Requirements for Rho/ROCK Signalling and Extracellular Proteolysis. Nat. Cell Biol. 5, 711–719. 10.1038/ncb1019 12844144

[B84] Sanz-Moreno V. Gadea G. Ahn J. Paterson H. Marra P. Pinner S. (2008). Rac Activation and Inactivation Control Plasticity of Tumor Cell Movement. Cell 135, 510–523. 10.1016/j.cell.2008.09.043 18984162

[B85] Sanz-Moreno V. Marshall C. J. (2010). The Plasticity of Cytoskeletal Dynamics Underlying Neoplastic Cell Migration. Curr. Opin. Cell Biol. 22, 690–696. 10.1016/j.ceb.2010.08.020 20829016

[B86] Sedzinski J. Biro M. Oswald A. Tinevez J.-Y. Salbreux G. Paluch E. (2011). Polar Actomyosin Contractility Destabilizes the Position of the Cytokinetic Furrow. Nature 476, 462–466. 10.1038/nature10286 21822289

[B87] Severson A. F. Baillie D. L. Bowerman B. (2002). A Formin Homology Protein and a Profilin Are Required for Cytokinesis and Arp2/3-independent Assembly of Cortical Microfilaments in *C. elegans* . Curr. Biol. 12, 2066–2075. 10.1016/s0960-9822(02)01355-6 12498681

[B88] Somlyo A. P. Somlyo A. V. (2003). Ca2+Sensitivity of Smooth Muscle and Nonmuscle Myosin II: Modulated by G Proteins, Kinases, and Myosin Phosphatase. Physiol. Rev. 83, 1325–1358. 10.1152/physrev.00023.2003 14506307

[B89] Srivastava N. Kay R. R. Kabla A. J. (2017). Method to Study Cell Migration under Uniaxial Compression. MBoC 28, 809–816. 10.1091/mbc.e16-08-0575 28122819 PMC5349787

[B90] Srivastava N. Traynor D. Piel M. Kabla A. J. Kay R. R. (2020). Pressure Sensing through Piezo Channels Controls whether Cells Migrate with Blebs or Pseudopods. Proc. Natl. Acad. Sci. U.S.A. 117, 2506–2512. 10.1073/pnas.1905730117 31964823 PMC7007555

[B91] Stockem W. Hoffmann H.-U. Gawlitta W. (1982). Spatial Organization and Fine Structure of the Cortical Filament Layer in Normal Locomoting Amoeba proteus. Cell Tissue Res. 221, 505–519. 10.1007/bf00215699 7198940

[B92] Sun T. J. Devreotes P. N. (1991). Gene Targeting of the Aggregation Stage cAMP Receptor cAR1 in Dictyostelium. Genes Dev. 5, 572–582. 10.1101/gad.5.4.572 1849108

[B93] Taddei M. L. Giannoni E. Comito G. Chiarugi P. (2013). Microenvironment and Tumor Cell Plasticity: An Easy Way Out. Cancer Lett. 341, 80–96. 10.1016/j.canlet.2013.01.042 23376253

[B94] Tinevez J.-Y. Schulze U. Salbreux G. Roensch J. Joanny J.-F. Paluch E. (2009). Role of Cortical Tension in Bleb Growth. Proc. Natl. Acad. Sci. U.S.A. 106, 18581–18586. 10.1073/pnas.0903353106 19846787 PMC2765453

[B95] Tournaviti S. Hannemann S. Terjung S. Kitzing T. M. Stegmayer C. Ritzerfeld J. (2007). SH4-domain-induced Plasma Membrane Dynamization Promotes Bleb-Associated Cell Motility. J. Cell Sci. 120, 3820–3829. 10.1242/jcs.011130 17959630

[B96] Tozluoğlu M. Tournier A. L. Jenkins R. P. Hooper S. Bates P. A. Sahai E. (2013). Matrix Geometry Determines Optimal Cancer Cell Migration Strategy and Modulates Response to Interventions. Nat. Cell Biol. 15, 751–762. 10.1038/ncb2775 23792690

[B97] Trinkaus J. P. (1973). Surface Activity and Locomotion of Fundulus Deep Cells during Blastula and Gastrula Stages. Dev. Biol. 30, 68–103. 10.1016/0012-1606(73)90049-3 4735370

[B98] Tsujioka M. Yumura S. Inouye K. Patel H. Ueda M. Yonemura S. (2012). Talin Couples the Actomyosin Cortex to the Plasma Membrane during Rear Retraction and Cytokinesis. Proc. Natl. Acad. Sci. U.S.A. 109, 12992–12997. 10.1073/pnas.1208296109 22826231 PMC3420178

[B99] Tyson R. A. Zatulovskiy E. Kay R. R. Bretschneider T. (2014). How Blebs and Pseudopods Cooperate during Chemotaxis. Proc. Natl. Acad. Sci. U.S.A. 111, 11703–11708. 10.1073/pnas.1322291111 25074921 PMC4136620

[B100] Ullo M. F. Logue J. S. (2021). ADF and Cofilin-1 Collaborate to Promote Cortical Actin Flow and the Leader Bleb-Based Migration of Confined Cells. Elife 10, e67856. 10.7554/elife.67856 34169836 PMC8253594

[B101] Venturini V. Pezzano F. Català Castro F. Häkkinen H.-M. Jiménez-Delgado S. Colomer-Rosell M. (2020). The Nucleus Measures Shape Changes for Cellular Proprioception to Control Dynamic Cell Behavior. Science 370, eaba2644. 10.1126/science.aba2644 33060331

[B102] Wang Y. Litvinov R. I. Chen X. Bach T. L. Lian L. Petrich B. G. (2008). Loss of PIP5KIγ, unlike Other PIP5KI Isoforms, Impairs the Integrity of the Membrane Cytoskeleton in Murine Megakaryocytes. J. Clin. Invest. 118, 812–819. 10.1172/jci34239 18188447 PMC2176194

[B103] Wolf K. Mazo I. Leung H. Engelke K. von Andrian U. H. Deryugina E. I. (2003). Compensation Mechanism in Tumor Cell Migration. J. Cell Biol. 160, 267–277. 10.1083/jcb.200209006 12527751 PMC2172637

[B104] Wong M. Gilmour D. (2021). Going Your Own Way: Self-Guidance Mechanisms in Cell Migration. Curr. Opin. Cell Biol. 72, 116–123. 10.1016/j.ceb.2021.07.004 34403875

[B105] Yoshida K. Inouye K. (2001). Myosin II-dependent Cylindrical Protrusions Induced by Quinine inDictyostelium: Antagonizing Effects of Actin Polymerization at the Leading Edge. J. Cell Sci. 114, 2155–2165. 10.1242/jcs.114.11.2155 11493651

[B106] Yoshida K. Soldati T. (2006). Dissection of Amoeboid Movement into Two Mechanically Distinct Modes. J. Cell Sci. 119, 3833–3844. 10.1242/jcs.03152 16926192

[B107] Zatulovskiy E. Tyson R. Bretschneider T. Kay R. R. (2014). Bleb-driven Chemotaxis of Dictyostelium Cells. J. Cell Biol. 204, 1027–1044. 10.1083/jcb.201306147 24616222 PMC3998804

[B108] Zhao S. Liao H. Ao M. Wu L. Zhang X. Chen Y. (2014). Fixation-induced Cell Blebbing on Spread Cells Inversely Correlates with Phosphatidylinositol 4,5-bisphosphate Level in the Plasma Membrane. Febs Open Bio 4, 190–199. 10.1016/j.fob.2014.02.003 PMC3953720 24649401

